# Broadband photon-photon interactions mediated by cold atoms in a photonic crystal fiber

**DOI:** 10.1038/srep25630

**Published:** 2016-05-12

**Authors:** Marina Litinskaya, Edoardo Tignone, Guido Pupillo

**Affiliations:** 1 icFRC, IPCMS (UMR 7504) and ISIS (UMR 7006), Université de Strasbourg and CNRS, 67000 Strasbourg, France

## Abstract

We demonstrate theoretically that photon-photon attraction can be engineered in the continuum of scattering states for pairs of photons propagating in a hollow-core photonic crystal fiber filled with cold atoms. The atoms are regularly spaced in an optical lattice configuration and the photons are resonantly tuned to an internal atomic transition. We show that the hard-core repulsion resulting from saturation of the atomic transitions induces bunching in the photonic component of the collective atom-photon modes (polaritons). Bunching is obtained in a frequency range as large as tens of GHz, and can be controlled by the inter-atomic separation. We provide a fully analytical explanation for this phenomenon by proving that correlations result from a mismatch of the quantization volumes for atomic excitations and photons in the continuum. Even stronger correlations can be observed for in-gap two-polariton bound states. Our theoretical results use parameters relevant for current experiments and suggest a simple and feasible way to induce interactions between photons.

There is growing interest in realising strongly interacting photons^1^,^2^ for applications in quantum information processing^3^,^4^,^5^,^6^,^7^,^8^,^9^,^10^, quantum metrology^11^,^12^,^13^ and many-body physics^14^,^15^,^16^,^17^. Photonic non-linearities are often induced by coupling photons to two-level emitters, as achieved in atomic and molecular setups with single emitters in a cavity configuration^18^,^19^. Alternatively, photonic non-linearities can result, e.g., from the anharmonicity of the multi-excitation spectra in the Jaynes-Cummings Hamiltonian^20^,^21^,^22^,^23^,^24^, or from dipolar or van-der-Waals interactions between atoms, such as in Rydberg atoms under condition of electromagnetically induced transparency (EIT)^25^,^26^,^27^,^28^,^29^. Recent groundbreaking experiments^28^,^29^, in particular, have demonstrated attraction of photons caused by formation of bound bipolariton states^30^,^31^.

In this Report we propose the observation of photon-photon interactions and bunching in an ordered ensemble of two-level atoms confined to one-dimension (1D) and resonantly coupled to the transverse photons of a cavity [Fig. 1(a)]. The interaction of atoms and light in the strong exciton-photon coupling regime results in the formation of a doublet of polaritonic modes [Fig. 1(b)], corresponding to coherent superpositions of photonic and collective atomic excitations (excitons). In our scheme the non-linearity results solely from the kinematic interaction between these excitons: The latter amounts to a hard-core repulsion and is due to atomic saturability, as one atom can accommodate at most one excitation.

The existence of kinematic interaction in solids has been known for decades^32^, however, was always considered as a very weak effect. In contrast, here we demonstrate that kinematic interaction in a cold atom setup may lead to a pronounced bunching in the photonic component of the coupled polaritonic states. As opposed to narrow bound state resonances in the MHz frequency range typical, e.g., of Rydberg atoms, this bunching appears in the continuum of unbound two-polariton states, and can be observed in a broad GHz frequency range for parameters within the reach of current experimental technologies.Via the exact solution for the subsystem consisting of excitons uncoupled from light, we demonstrate that the bunching is the result of the mismatch of the quantization volumes for states with (excitons) and without (photons) hard core repulsion. Due to the broadband nature of the effect, this type of non-linearity is expected to be comparatively resilient against decoherence. We conclude by discussing the occurrence of bound two-polariton states within spectral gaps.

The scheme we have in mind consists of two-level atoms trapped in a 1D optical lattice in the Mott insulator state (i.e. with one atom per lattice site), and confined inside a 1D resonant cavity. For concreteness, in this work we consider the D2-line of Rb atoms placed into a hollow-core photonic crystal fiber – as in the experiment^33^ with Sr. If the cavity losses are low and atoms are well-ordered, in this geometry one can work near the atomic transition without imposing the EIT condition to eliminate absorption: Polaritonic states are coherent superpositions of photonic and collective atomic excitations, as opposed to incoherent absorption of individual atoms. Besides of the hollow-core fibers^8^,^33^,^34^,^35^,^36^,^37^,^38^,^39^, one can think of other implementations of this scheme, as recent theoretical studies have investigated a variety of systems that allow for coupling photons to an ensemble of two-level emitters in a 1D configuration^40^,^41^,^42^,^43^,^44^,^45^,^46^. Solid-state realizations are also possible, e.g., using Si vacancies in photonic crystals^47^,^48^.

## Results

### Model

The setup consists of *N* atoms trapped on a lattice and coupled to a cavity. Its Hamiltonian is


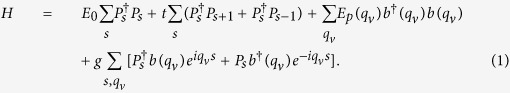


Here, *P*_*s*_, 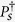 and *b*(*q*_*ν*_), *b*^†^(*q*_*ν*_) destroy and create an atomic excitation at site *s* and a photon with the wave vector *q*_*ν*_ along the cavity axis, respectively; 
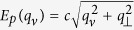
 is the photon energy, *q*_*ν*_ = 2*πν*/(*Na*) with integer index *ν* ∈ (−*N*/2, *N*/2] (*a* is the inter-particle spacing, *c* is the speed of light), and *q*_⊥_ is the transverse photon momentum [for the lowest mode of a perfect open cylindrical resonator of radius *R*, it is found from the first zero of the function *J*_0_(*q*_⊥_*R*)]; *E*_0_ is the atomic transition frequency, *t* ∝ *d*^2^/*a*^3^ is the hopping energy for the atomic excitations in the nearest neighbor approximation, 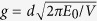 is the atom-light coupling constant, with *d* the transition dipole moment and *V* = *πR*^2^*Na* the volume of the cavity. The atomic part of equation (1) is readily diagonalized by Frenkel exciton operators^32^ 
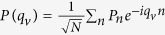
 describing extended wave functions resulting from exciton hopping. Although the hopping term in the Hamiltonian is typically much weaker than the light-matter coupling (*t* ≪ *g*), we keep it as it is important to properly describe the polaritonic dispersion at large momenta, where the polariton behaves as a bare exciton.

In the following, we solve the Schrödinger equation in the two-particle subspace, where a wave function reads


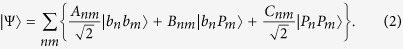


Here, 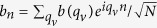, while 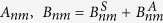 and *C*_*nm*_ are the amplitudes for finding two relevant states (photons or atomic excitations) at sites *n*, *m* (the superscripts *S*, *A* stay for symmetric/antisymmetric; the amplitudes *A* and *C* are always symmetric). We recast the Schrödinger equation as a set of equations for *A*, *B* and *C*, and solve it in terms of total and relative wave vectors of two particles, 
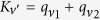
 and 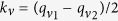, respectively, where only *K*_*ν*′_ is a good quantum number. Here, 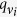 indicates the wave vector of the first (*i* = 1) and second (*i* = 2) particle; *K*_*ν*′_ = 4*πν*′/(*Na*) with *ν*′ ∈ (−*N*/2, *N*/2] and *k*_*ν*_ = 2*πν*/(*Na*) with *ν* ∈ (- *N*/2, *N*/2). Below we consider in detail the case *K*_*ν*′_ = 0, and briefly discuss *K*_*ν*′_ ≠ 0. For *K*_*ν*′_ = 0, 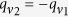, and *k*_*ν*_ = 2*πν*/(*Na*) with an integer index *ν* ∈ (−*N*/2, *N*/2] (a detailed discussion of the quantum numbers describing *K* and *k* for bosons on a lattice is provided e.g., in Ref. 49). We obtain:


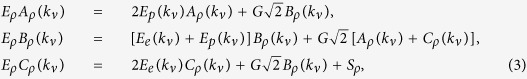


where *E*_*e*_(*k*_*ν*_) = *E*_0_ + 2*t* cos *ak*_*ν*_ is the exciton energy, *ρ* labels the two-polariton state in the order of increasing energy (we note that several energies *E*_*ρ*_ correspond to a same wave vector *k*_*ν*_, one for each two-polariton band), and


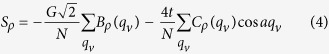


is a *k*_*ν*_-independent term accounting for polariton-polariton scattering due to the kinematic interaction; *B* = *B*^*S*^ and *B*^*A*^ = 0 for *K*_*ν*′_ = 0; 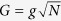 is the collective atom-light coupling constant.

For *S*_*ρ*_ = 0, equation (3) describes non-interacting polaritons with dispersion determined by the condition


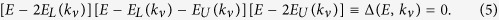


Here, *E*_*L*_(*k*_*ν*_) and *E*_*U*_(*k*_*ν*_) are the energies of the lower (*L*) and upper (*U*) polaritons, respectively, with


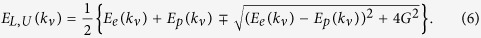


**Figure 1:**
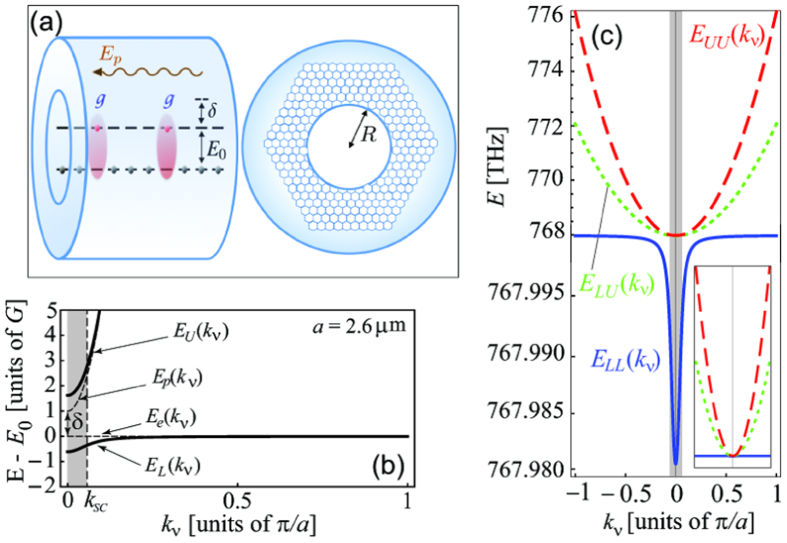
(**a**) Sketch of the two-level atomic ensemble embedded into a cylindrical cavity showing main parameters (see text). (**b**) Dispersion of lower and upper polaritons, *E*_*L*_(*k*_*ν*_) and *E*_*U*_(*k*_*ν*_), vs. those of uncoupled exciton and cavity photon, *E*_*e*_(*k*_*ν*_) and *E*_*p*_(*k*_*ν*_), with positive detuning *δ* = *E*_*p*_(0) − *E*_0_. Shade marks the strong coupling region approximately restricted by *k*_*SC*_. (**c**) Non-interacting two-polariton states: *E*_*LL*_(*k*_*ν*_) = 2*E*_*L*_(*k*_*ν*_), *E*_*LU*_(*k*_*ν*_) = *E*_*L*_(*k*_*ν*_) + *E*_*U*_(*k*_*ν*_), *E*_*UU*_(*k*_*ν*_) = 2*E*_*U*_(*k*_*ν*_), *a* = 2.66 *μ*m, *δ* = 0. Note different energy scales for lower and upper halves of the plot. Inset shows three two-polariton branches plotted together at the same energy scale.

Figure 1(b) shows that the Brillouin zone can be roughly divided into two distinct regions: The strong-coupling region with *k*_*ν*_ < *k*_*SC*_ near the atom-photon resonance [shaded region in Fig. 1(b,c)], and the region with *k*_*ν*_ > *k*_*SC*_, where polaritons essentially behave as uncoupled exciton and photon. The characteristic wave vector 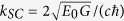 is determined by the condition *E*_*L*_(*k*_*SC*_) = *E*_0_ with the parabolic approximation for *E*_*L*_(*k*_*ν*_) valid for small *k*_*ν*_.

For finite kinematic interaction *S*_*ρ*_ ≠ 0 and the solutions of equation (3) are wave packets of free-polariton states. Below we demonstrate that correlations between photons arise as a result of constructive interference among several components of these wave packets. This effect is more prominent the larger the strong coupling region. In the following, we explain it by first solving analytically the Schrödinger equation for bare excitons uncoupled from photons, and then by describing the effects of strong coupling of excitons to photons. The latter results in photonic bunching in a broad frequency range in the continuum. The existence of bound two-photon states within polaritonic gaps is discussed towards the end of the work.

### Two-photon correlations

Equations (3) can be solved analytically. By expressing the amplitudes *A*, *B* and *C* through each other and using the equality 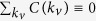, which follows from the kinematic interaction constraint 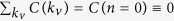 (*n* is the relative distance between two excitations in the site representation), we reduce it to three independent equations of the form


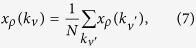


with *x*_*ρ*_(*k*_*ν*_) staying for *A*_*ρ*_(*k*_*ν*_)Δ(*E*_*ρ*_, *k*_*ν*_), *B*_*ρ*_(*k*_*ν*_)Δ(*E*_*ρ*_, *k*_*ν*_)/(*E*_*ρ*_ − 2*E*_*p*_(*k*_*ν*_)) and *C*_*ρ*_(*k*_*ν*_)Δ(*E*_*ρ*_, *k*_*ν*_)/*ϕ*(*E*_*ρ*_, *k*_*ν*_), where Δ(*E*_*ρ*_, *k*_*ν*_) is defined in equation (5), *ϕ*(*E*, *k*_*ν*_) = [*E* − 2*E*_*p*_(*k*_*ν*_)][*E* − *E*_*p*_(*k*_*ν*_) − *E*_*e*_(*k*_*ν*_)] − 2*G*^2^. The left-hand side of Eq. (7) depends on *k*_*ν*_, while the right-hand side does not. This basically means that Eq. (7) is solved by *x*_*ρ*_(*k*_*ν*_) ≡ *x*_*ρ*_ = *const* (*ρ*). Introducing the normalization constant


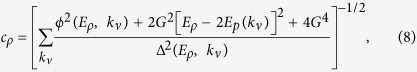


we finally write


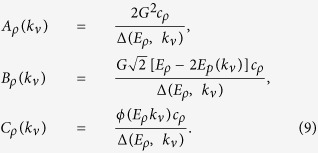


**Figure 2:**
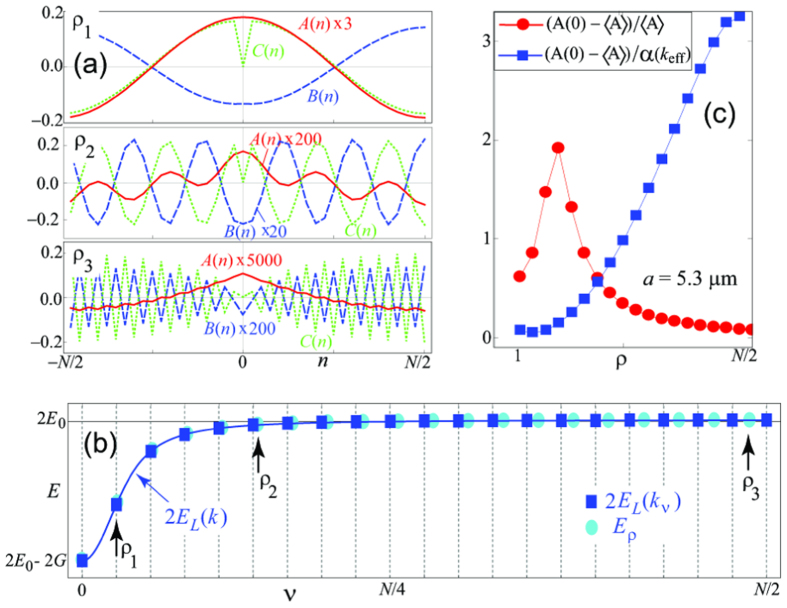
(**a**) Amplitudes *X*(*n*) − 〈*X*(*n*)〉, *X* = *A* (red), *B* (blue dashed) and *C* (green dotted), scaled by the factors shown in each figure, for three two-polariton states marked by arrows in panel (**b**); *n* is the relative distance, *N* = 40, *a* = 5.3 *μ*m. (**b**) Exact two-polariton energies (cyan), compared to the energies of non-interacting polaritons 2*E*_*L*_(*k*_*ν*_) (blue squares). Cyan points are plotted at the positions given by *k*_eff_(*ρ*).(**c**) Bunching strength as function of state number, absolute value (red circles) and scaled with the account of photonic amplitude of the state (blue squares).

Figure 2(a) shows example results for the real-space Fourier transform of the amplitudes (9) for the three states from the bottom, middle and top of the two-polariton lower-lower- (LL-) band, whose energies are indicated by arrows in Fig. 2(b). For small *ρ* (upper plot) all amplitudes resemble free wave states with a sharp dip in the two-exciton amplitude *C*(*n*) at *n* = 0, which is a result of the hard-core constraint. With the increase of *ρ* within the LL-band, the amplitudes *B*(*n*) and *C*(*n*) demonstrate modulated oscillations, with the excitation probability increasing towards larger separations. In contrast, the two-photon amplitude *A*(*n*) stops oscillating for larger *ρ* and displays a peak-like feature centered at *n* = 0 (see lower plot): The two-photon amplitude thus demonstrates bunching in the presence of repulsive kinematic interaction among atomic excitations.

In order to clarify the behavior of the amplitudes, we first solve the Schrödinger equation


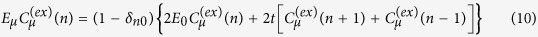


for two bare excitons interacting via the kinematic interaction in the nearest neighbor approximation (i.e., in the absence of coupling to photons). Remarkably, we find that this latter equation is analytically solvable (see Supplementary Materials for details). Due to the exclusion of the state *C*^(*ex*)^(*n* = 0) caused by the kinematic interaction, the states are now described by a new set of wave vectors {*κ*_*μ*_}, with


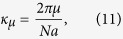


and 
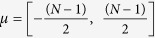
 *half-integer*. The set {*κ*_*μ*_} has elements that lie exactly between those of the original set {*k*_*ν*_}. This suggests that the exciton-exciton kinematic interaction, however weak, is a non-perturbative effect. The new two-exciton eigenenergies are then 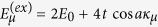, and their amplitudes read


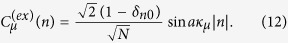


For finite exciton-photon coupling and 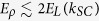, the exciton-photon coupling prevails over exciton-exciton interactions. In this regime each eigenstate is to a good approximation described by a single *k*_*ν*_, and the *C*-amplitudes behave approximately as *C*_*ν*_(*n*) ∝ (1 − *δ*_*n*0_)cos *ank*_*ν*_, i.e. as the symmetric part of a plane wave with 2*ν* nodes. For 
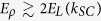
, however, the exciton-exciton interaction prevails over light-matter coupling, and the amplitudes *C* reach the exciton-like limit, where they are well approximated by equation (12). In other words, with the increase of *ρ*, the two-polariton quantum number *ρ* makes a smooth transition from *ν*-numbers to *μ*-numbers. We find that in all parameter regimes the two-polariton energy is well approximated by the analytical expression *E*_*ρ*_ = 2*E*_*L*_(*k*_eff_(*ρ*)), where


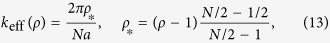


correspond to an effective wave vector *k*_eff_ and its label 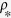, respectively, interpolating between the two sets above. This is shown in Fig. 2(b), where the exact numerical results for the energies from equation (3) (cyan dots) plotted as a function of the effective wave vectors *k*_eff_ perfectly match the analytical estimates *E*_*LL*_(*k*_*ν*_ → *k*_eff_(*ρ*)).

In the basis of the original wave vector set {*k*_*ν*_}, the gradual shift to the set {*κ*_*μ*_} corresponds to the formation of wave packets. The resonant character of the factor 1/Δ(*E*_*ρ*_, *k*_*ν*_) in equation (9) implies that *A*_*ρ*_(*k*_*ν*_) is peaked at *k*_*ν*_ ~±*k*_eff_(*ρ*). However, these components dominate the shape of *A*_*ρ*_(*k*_*ν*_) only for states within the strong coupling region, i.e. when *k*_eff_(*ρ*) < *k*_*SC*_. In contrast, for *k*_eff_(*ρ*) > *k*_*SC*_, the components *A*_*ρ*_(*k*_*ν*_ ~ *k*_eff_(*ρ*)) are strongly suppressed, as 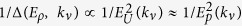 decays fast outside of the strong coupling region [see inset in Fig. 1(c)]. Therefore, for larger *ρ* only low-*k*_*ν*_ states (with 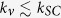) are found to contribute to *A*_*ρ*_(*k*_*ν*_); in other words, higher-*k*_*ν*_ states are too off-resonant to participate in the formation of the wave packets and, as a result, at large *ρ* the amplitudes *A*(*k*) develop a single maximum around *k*_*ν*_ = 0. This cusp-like structure of *A*_*ρ*_(*k*_*ν*_) results in the cusp-like shape of *A*_*ρ*_(*n*) in real space [Fig. 2(a), middle and lower panels]. This explains the central result of this paper: The mismatch between the quantum numbers describing the interacting (*C*) and non-interacting (*A*–*B*) subsystems leads to systematic two-photon bunching at *n* = 0. As *A*_*ρ*_(*n*) takes its maximum value at the same separation *n* = 0 for all *ρ* states with 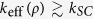, we expect that it should not be averaged out by a finite width of the exciting source. In the Supplementary Materials, we quantify these arguments and interpret the effect in terms of interference between different *A*_*ρ*_(*k*_*ν*_)-components.

### Controlling two-photon correlations

Correlations in the continuum of two-polariton states should be observable with current experimental technologies with Rb or Sr atoms in a Mott insulator state with unit filling, placed in a hollow-core fiber, e.g., in the configuration of Okaba *et al.*^33^. For example, choosing the radius of the fiber *R* = 0.299 *μ*m, we bring the lowest cavity mode in resonance with the D2 transition in Rb (transition dipole *d* = 4.22 a.u.) at *E*_0_ = 384 THz. We quantify the two-photon bunching by the figure of merit Δ*A*_*ρ*_ defined as


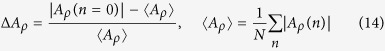


if the difference is positive, and zero otherwise and plot it in Fig. 2(c) as a function of the state index *ρ* (red circles). The apparent decrease of Δ*A* for large *ρ* is a result of the overall decrease of the photonic wave function in the polaritonic state. To demonstrate that the bunching effect in fact *increases* with increasing *ρ*, in Fig. 2(c) we plot (*A*_*ρ*_(0) − 〈*A*_*ρ*_〉)/*α*(*k*_eff_) (blue squares), where 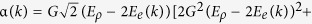 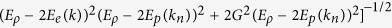 is the two-photon amplitude in the absence of interactions.

**Figure 3:**
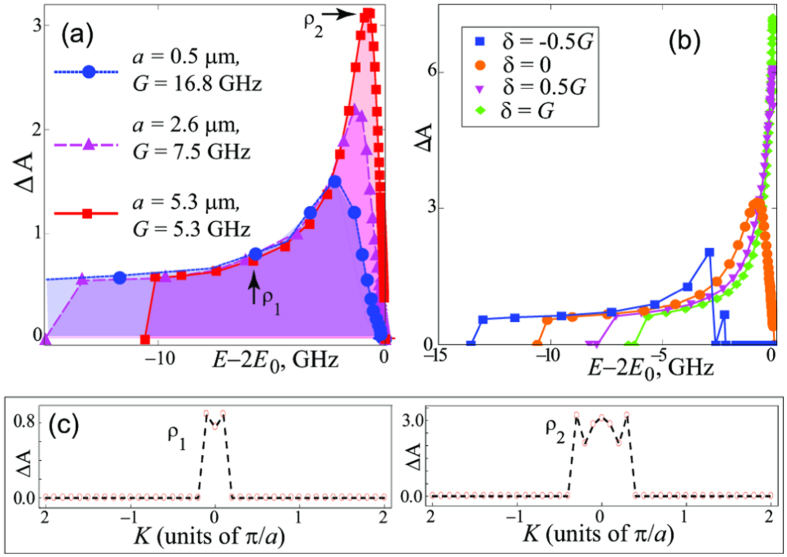
(**a**) Figure of merit Δ*A* as a function of the state energy for Rb atoms, D2 line, for *a* = 532 nm, 2.66 *μ*m and 5.32 *μ*m. (**b**) Δ*A* in the LL-band for *a* = 5.3 *μ*m for different values of detuning. **(c)** Δ*A* as function of *K* for two states, which at *K*_*ν*′_ = 0 correspond to the states marked by *ρ*_1_ and *ρ*_2_ in panel (**a**); *a* = 5.3 *μ*m.

Figure 3(a) shows that Δ*A*(*E*_*ρ*_) changes by varying the lattice constant *a* from 532 nm to 5.32 *μ*m. Counterintuitively, Δ*A* is found to *increase* for larger *a*, corresponding to lower atomic density. This is explained by noting that only states within the strong coupling region, which have both finite two-exciton amplitude (to interact effectively) and finite two-photon amplitude (the observable), contribute to *A*_*ρ*_(*n* = 0). Therefore, photon bunching is most pronounced when *k*_*SC*_ is comparable to the size of the Brillouin zone, which can be easily achieved in cold atom experiments. The latter condition requires larger inter-atomic separations, as *ak*_*SC*_/*π* ∝ *a*^3/4^. This also explains why this type of bunching of continuum states cannot be observed in solids: For electronic transitions of ~2 eV and typical lattice constants *a *~ 5 Å, the relative size of the strong coupling region 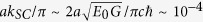. On the contrary, Fig. 3(a) shows that in cold atom systems considerable bunching can be realised in a continuous band of the order of several GHz.

In Fig. 3(b) we examine Δ*A* vs energy for a few values of the detuning *δ* = *E*_*p*_(0) − *E*_0_ between the cavity mode *E*_*p*_(0) and the excitonic resonance *E*_0_, by varying *δ* between −*G*/2 and *G*. We find that large negative values of *δ* result in wider bunching frequency intervals due to wider *E*_*LL*_ bands.

Finally, it is of crucial importance that the bunching survives for states with *K*_*ν*′_ ≠ 0. The latter correspond to propagation of the center of mass of the two polaritons, and can be directly observed in experiments. The equations for the amplitudes at *K*_*ν*′_ ≠ 0 are bulky and will be published elsewhere. Here we only demonstrate the existence of bunching in a wide range of 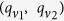-pairs by plotting in Fig. 3(c) Δ*A* as a function of *K*_*ν*′_ for two states, selected so that at *K*_*ν*′_ = 0 they correspond to the full squares marked as *ρ*_1_ and *ρ*_2_ in Fig. 3(a).

### Gap states

**Figure 4:**
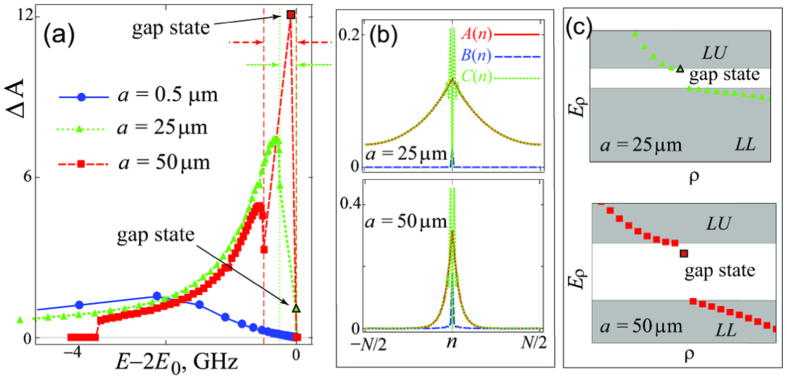
(**a**) Figure of merit for bunching Δ*A* as a function of the state energy for Rb atoms, D2 line, for *a* = 0.5 *μ*m, 25 *μ*m and 50 *μ*m. The vertical green dotted (red dashed) line marked by a green dotted (red dashed) right-pointing arrow defines the lower boundary of the gap for *a* = 25 *μ*m (*a* = 50 *μ*m). The two left-pointing matching arrows mark the corresponding upper boundaries. The two symbols with black contours indicate gap bipolaritons. Panel (**b**) shows the amplitudes *A*(*n*), *B*(*n*) and *C*(*n*) for the gap states of panel (**a**). Panel (**c**) shows the energies *E*_*ρ*_ as a function of the state number *ρ* for the states of panel (**a**) which are located near the gap region (gray shaded areas indicate the LL- and LU- bands). At *a* ~ 25 *μ*m the lowest LU-state already has a typical bound state wave function, but is situated extremely close to LU-band. At *a* ~ 50 *μ*m the lowest LU-state is a true gap bipolariton.

A gap in the polaritonic spectrum appears naturally when the detuning is positive. In contrast, for *δ* = 0 and small *a* the two-polariton spectrum is non-gapped, as the energy for two lower polaritons *E*_*LL*_(*π*/*a*) equals *E*_*LL*_(*π*/*a*) = *E*_*LU*_(0) = 2*E*_0_, with *E*_*LU*_ the energy of the lower-upper- (LU-) band. However if *a* is so large that *k*_*SC*_ ~ *π*/*a*, then *E*_*LL*_(*π*/*a*) < 2*E*_0_ as the lower polariton does not reach the excitonic limit, and a small gap Δ_*LU*_ = *E*_*LU*_(*k*_*ν*_ = 0) − *E*_*LL*_(*k*_*ν*_ = *π*/*a*) opens between the LL- and LU- bands at zero detuning. In this regime one can observe the formation of a bunched state also *within the gapped region* in a close proximity of the LU-band, see Fig. 4(a). Contrary to the discussion above, this is a *polariton-polariton bound state* with very distinct wave functions, with *A*- and *B*-amplitudes peaked around *n* = 0 (the two-exciton *C*-component vanishes at *n* = 0 and therefore is maximal for the separation |*n*| = 1, in accordance with the hard core restriction) [see Fig. 4(b)]. For moderate *a*, the bound state merges with the LU-band. With the increase of the lattice constant, starting from *a* ~ 25 *μ*m, the bound state gradually splits from the band and shifts into the gap. Further increase of *a* is accompanied by deeper penetration into the gap [see Fig. 4(c)] and dramatic increase of the photonic bunching in this particular state, as shown in Fig. 4(a). The magnitude of the photonic bunching in this bound state is of the same order of magnitude as that one demonstrated by recent experiments employing Rydberg atoms^29^. We notice that while the photon-to-exciton ratio, 〈*A*_*ρ*_〉/〈*C*_*ρ*_〉, in high-momentum scattering states in the LL-band is significantly smaller compared to that of Coulomb-like two-photon states in Rydberg platforms^31^, the scattering states can display a bunching of similar strength as the in-gap bipolariton [see Fig. 4(a)], which is one of the main results of the paper.

The state above is a gap bipolariton forming under repulsive kinematic interaction. This is similar to the kinematic biexciton appearing in organic crystals with two molecules in a unit cell^50^. However, there the kinematic biexciton overlaps with the continuum band, and can be easily destroyed by, e.g., disorder or coupling to phonons. In contrast, the kinematic bipolariton described here is located in the gap and is thus stable against decoherence. Other types of bound states, both below the LL-continuum and in the polariton gap, can form if the atoms interact via, e.g., dipole forces. The gap states of both types are analogous to, e.g., the gap bound states found in atomic systems^51^,^52^ and Jaynes-Cummings-type models with repulsive interactions^53^.

For *a* large enough the interaction of excitons with photons from higher Brillouin zones becomes possible: An exciton with a wave vector *k*_*ν*_ is coupled not only to a photon with the same *k*_*ν*_ but also to photons with *k*_*ν*_ ± 2*π*/*a*, *k*_*ν*_ ± 4*π*/*a*, etc. For the setup described here, this occurs for *a* ≳ 50 *μ*m. What happens in this regime will be the subject of a further investigation.

## Discussion

In this article we have characterised the correlations that are generated for pairs of 1D photons propagating in a hollow-core crystal fiber and coupled to an ordered atomic ensemble. The discussed two-photon correlations appear to be of two distinct types: (*i*) Those resulting from the bound state in the gap and (*ii*) those arising from the scattering states. With respect to (*i*), we have shown that the bipolariton state localized in the gap exhibits strong photon-photon correlations, similar to those observed by Firstenberg, O. *et al.*^29^. With respect to (*ii*) we have found that the magnitude of correlations are of the same order of magnitude as those of type (*i*). This two-photon bunching can be observed in GHz frequency window, which greatly exceeds MHz frequency intervals typical for the correlations induced by, e.g., three-level cold Rydberg atoms in the EIT regime, and can be controlled by tuning the inter-atomic spacing. Our results are valid as long as the polaritonic splitting 2*G* exceeds strongly the sum of the excitonic and photonic broadenings, which can be achieved by using a high-quality cavity and by careful preparation of the atomic Mott insulator state. Other realizations of this scheme are also possible, as long as broadenings can be kept smaller than the collective exciton-photon coupling. In addition to 1D hollow-core crystal fibers, promising candidates are metallic nanowires^54^ and nanophotonic waveguides^15^,^55^,^56^,^57^,^58^,^59^,^60^.

The same effect as demonstrated here, and even in an exaggerated form, can exist for chains of two-level Rydberg atoms coupled to 1D cavity photons. We find that the formation of a large-radius Rydberg blockade sphere considerably enhances bunching, as the effect of the quantization volumes mismatch presented in our work becomes more pronounced. In addition, the long-range dynamical (dipole-dipole or van der Waals) interaction is found to enrich the arising non-linear effects. These results will be subject of a forthcoming publication.

## Additional Information

**How to cite this article**: Litinskaya, M. *et al.* Broadband photon-photon interactions mediated by cold atoms in a photonic crystal fiber. *Sci. Rep.* **6**, 25630; doi: 10.1038/srep25630 (2016).

## Supplementary Material

Supplementary Information
