# Supplementary material for: Broadband photon-photon interactions mediated by cold atoms in a photonic crystal fiber
Source: Sci Rep. 2016 May 12;6:25630. doi: 10.1038/srep25630 (PMC4864373; doi:10.1038/srep25630)
Supplement: Supplementary Information [file srep25630-s1.pdf]

SUPPLEMENTARY MATERIAL TO
"Broadband photon-photon interactions mediated by cold atoms in a photonic crystal fiber"

Marina Litinskaya, Edoardo Tignone, Guido Pupillo

Kinematic interaction for bare excitons

The Schrödinger equation for two bare excitons ($G \equiv 0$) on sites n_1 and n_2 interacting via kinematic interaction is

$$EC^{(ex)}_{n_1n_2} = 2E_0C^{(ex)}_{n_1n_2} + (1-\delta_{n_1n_2})\sum_s \left(t_{n_1s}C^{(ex)}_{sn_2} + t_{n_2s}C^{(ex)}_{n_1s}\right) \tag{S1}$$

with t_{ij} the long-range hopping energy, and the same-site amplitude chosen as $C^{(ex)}_{nn} = 0$ [1]. Let n be $n = |n_1 - n_2|$, and the index μ enumerate the eigenstates of this equation. We rewrite equation (S1) in the nearest neighbour approximation:

$$E^{(ex)}_\mu C^{(ex)}_\mu(n) = (1-\delta_{n0})\left\{2E_0C^{(ex)}_\mu(n) + 2t[C^{(ex)}_\mu(n+1) + C^{(ex)}_\mu(n-1)]\right\}. \tag{S2}$$

One can verify that the normalized amplitudes which satisfy equation (S2) are

$$C^{(ex)}_\mu(n) \equiv g_n(\mu) = \frac{\sqrt{2}(1-\delta_{n0})}{\sqrt{N}}\ \sin a|n|\kappa_\mu \tag{S3}$$

with wave vectors $\kappa_\mu = 2\pi\mu/(Na)$ with a half-integer state index $\mu \in [-(N-1)/2, (N-1)/2]$ introduced in equation (10) in the main text. The basis functions $g_n(\mu)$ form an orthonormal set in both spaces, with the orthonormality conditions reflecting the permutation symmetry and the hard-core condition:

$$\begin{aligned} \textstyle\sum_n g_n(\mu_1)g_n(\mu_2) &= \delta_{|\mu_1|,|\mu_2|}, \\ \textstyle\sum_\mu g_{n_1}(\mu)g_{n_2}(\mu) &= (1-\delta_{n_10})\delta_{|n_1|,|n_2|}. \end{aligned} \tag{S4}$$

The eigenenergies of two-exciton states are also described by the wave vectors κ_μ as

$$E^{(ex)}_\mu = 2E_0 + 4t\ \cos a\kappa_\mu. \tag{S5}$$

As the new wave vectors κ_μ have a half-integer state index they lie exactly between the positions of the standard wave vectors k_ν for non-interacting excitons. As a consequence, the amplitudes $C^{(ex)}_\mu(k_\nu)$ do not have poles, but rather enhanced components $k_\nu \approx \kappa_\mu$, as can be seen from the Fourier transform of equation (S3):

$$C^{(ex)}_\mu(k) = \frac{\sin a\kappa_\mu\cos(ak_\nu) + (-1)^\mu \sin ak_\nu \sin(ak_\nu N/2)}{\cos ak_\nu - \cos a\kappa_\mu}. \tag{S6}$$

We conclude that the kinematic interaction is a weak, but absolutely non-perturbative effect for excitons. Let us now discuss the effect of the kinematic interaction for polaritons.

Creation of the wave packets

Two-polariton states in the presence of kinematic interaction can be viewed as composed of two subsystems: the non-interacting subsystem (consisting of photon-photon and photon-exciton states) is described by the quantum numbers $k_\nu = 2\pi\nu/(Na)$ with integer ν, whereas the interacting subsystem (consisting of exciton-exciton states) is described by $\kappa_\mu = 2\pi\mu/(Na)$ with half-integer μ. In the following discussion we shall stress the role of the two wave vector sets, which will be reflected in the adopted notations. The coupling between these two subsystems is responsible for intermixing the corresponding wave vector sets $\{k_\nu\}$ and $\{\kappa_\mu\}$ and eventually leads to the creation of the wave packets in the "original" wave vector set $\{k_\nu\}$. In particular, at the lowest energies the coupling of excitons to photons dominates over exciton-exciton interaction, and the corresponding polaritons are better described by k_ν. With the increase of the state number, instead, polaritons enter the exciton-like regime and are better described by κ_μ.

We introduce the operators $\alpha_n^\dagger$, $\beta_n^\dagger$ and $\gamma_n^\dagger$, which describe, respectively, creation of two photons, one photon and one exciton, and two excitons separated by a distance n. The two particle wave function takes the form $|\Psi\rangle = \sum_s \left[A(s)\,|\alpha_s\rangle + B(s)\,|\beta_s\rangle + C(s)\,|\gamma_s\rangle\right]$, and the Hamiltonian $\tilde{H}_{\rm eff} = \tilde{H}_{AB} + \tilde{H}_C^{(KI)} + \tilde{H}_{AB-C}^{(KI)}$ is made up of three terms:

$$\tilde{H}_{AB} = \sum_{n,m} \left[2E_p(n-m)\alpha_n^\dagger\alpha_m + \left(E_p(n-m)+E_e(n-m)\right)\beta_n^\dagger\beta_m\right] + G\sqrt{2}\sum\nolimits_n \left[\alpha_n^\dagger\beta_n + \beta_n^\dagger\alpha_n\right],$$

$$\tilde{H}_C^{(KI)} = \sum_{n,m}(1-\delta_{n0})2E_e(n-m)\gamma_n^\dagger\gamma_m, \tag{S7}$$

$$\tilde{H}_{AB-C}^{(KI)} = G\sqrt{2}\sum_n(1-\delta_{n0})\left[\gamma_n^\dagger\beta_n + \beta_n^\dagger\gamma_n\right],$$

where the last one describes the coupling between the interacting (C) and non-interacting (AB) subsystems. The resulting Schrödinger equation is identical to the Fourier transform of equations (3) in the main text.

The first term $\tilde{H}_{AB}$ describes the subspace "photon-photon $\bigcup$ photon-exciton", and is diagonalized by the operators ${\xi_\nu^{(i)}}^\dagger = X_\nu^{(i,\alpha)}\alpha_\nu^\dagger + X_\nu^{(i,\beta)}\beta_\nu^\dagger$:

$$\tilde{H}_{AB} = \sum_{i=L,U}\sum_\nu E_\nu^{(p,i)}{\xi_\nu^{(i)}}^\dagger\xi_\nu^{(i)}. \tag{S8}$$

Here and below $i=(U,L)$ is the index of the polaritonic branch, $\nu \in (-N/2, N/2]$ is the free-state wave index, and

$$E_\nu^{(p,i)} = E_\nu^{(p)} + \frac{E_\nu^{(e)} + E_\nu^{(p)} \pm \sqrt{\left(E_\nu^{(p)} - E_\nu^{(e)}\right)^2 + 8G^2}}{2} \tag{S9}$$

with $E_\nu^{(p)} \equiv E_p(k_\nu)$, $E_\nu^{(e)} \equiv E_e(k_\nu)$. The energies $E_\nu^{(p,i=\{L,U\})}$ (with $i=L$ corresponding to "$-$", and $i=U$ to "+" in the right-hand side) are constructed as sums of energies of one photon and one exciton-polariton with the coupling constant $\sqrt{2}G$, taken at the same wave vector k_ν. They naturally appear as solutions of the first two lines of equations (3) in the main text with $C\equiv 0$. The photon-photon and photon-exciton amplitudes are

$$X_\nu^{(i,\alpha)} = \sqrt{\frac{\left(E_\nu^{(p,i)} - E_\nu^{(p)} - E_\nu^{(e)}\right)^2}{2G^2 + \left(E_\nu^{(p,i)} - E_\nu^{(p)} - E_\nu^{(e)}\right)^2}}, \qquad X_\nu^{(i,\beta)} = \sqrt{1 - \left(X_\nu^{(i,\alpha)}\right)^2}. \tag{S10}$$

The two-exciton part $\tilde{H}_C^{(KI)}$ of the Hamiltonian is instead diagonalized as

$$\tilde{H}_C^{(KI)} = \sum_\mu E_\mu^{(ex)}\chi_\mu^\dagger\chi_\mu, \tag{S11}$$

with energy $E_\mu^{(ex)}$ defined in equation (S5), and

$$\chi_\mu^\dagger = \sum_{s=-N/2+1}^{N/2} g_s(\mu)\gamma_s^\dagger. \tag{S12}$$

Eventually, the interaction Hamiltonian $\hat{H}_{AB-C}$ can be rewritten in terms of ξ- and χ-operators as

$$\tilde{H}_{AB-C}^{(KI)} = \frac{G}{N}\sum_{i=L,U}\sum_{\nu\mu}\Lambda_{\nu\mu}X_\nu^{(i,\beta)}\left(\chi_\mu^\dagger\xi_\nu^{(i)} + {\xi_\nu^{(i)}}^\dagger\chi_\mu\right) \tag{S13}$$

with coupling coefficients $\Lambda_{\nu\mu}$ given by

$$\Lambda_{\nu\mu} = \frac{1}{2}\left[\cot\frac{\pi(\nu+|\mu|)}{N} - \cot\frac{\pi(\nu-|\mu|)}{N}\right]. \tag{S14}$$

The coefficients $\Lambda_{\nu\mu}$ intermix the wave vector sets k_ν and κ_μ.

The Schrödinger equation for the Hamiltonian $\tilde{H}_{\rm eff}$ and the wave function $|\Psi\rangle = \sum_{i\nu} p_\nu^{(i)} |\xi_\nu^{(i)}\rangle + \sum_\mu e_\mu |\chi_\mu\rangle$ leads to

$$\left(E - E_\nu^{(p,i)}\right) p_\nu^{(i)} = \frac{G X_\nu^{(i,\beta)}}{N} \sum_\mu \Lambda_{\nu\mu} e_\mu, \qquad \left(E - E_\mu^{(ex)}\right) e_\mu = \frac{G}{N} \sum_{i=L,U} \sum_\nu X_\nu^{(i,\beta)} \Lambda_{\nu\mu} p_\nu^{(i)}. \tag{S15}$$

We can exclude the exciton-exciton amplitudes e_μ from equations (S15); in the absence of hopping ($t \equiv 0$) the resulting equation for $p_{i\nu}$ reduces to

$$\left(E - E_\nu^{(p,i)}\right) p_\nu^{(i)} = \frac{G^2 X_\nu^{(i,\beta)}}{2N(E - 2E_0)} \sum_{i'=L,U} \sum_{\nu'} F_{\nu\nu'} X_{\nu'}^{(i',\beta)} p_{\nu'}^{(i')} \tag{S16}$$

with the kernel

$$F_{\nu\nu'} = N\left(\delta_{\nu,\nu'} + \delta_{\nu,-\nu'}\right) - \frac{2}{N}. \tag{S17}$$

The first term in the right-hand side of this equation describes the wave-vector-conserving scattering, while the second describes the formation of wave packets via scattering of non-interacting subsystem through the interacting one.

Within these notations, the amplitude for two photons being separated by n lattice sites is

$$A(n) = \langle \alpha_n | \Psi \rangle = \frac{1}{\sqrt{N}} \sum_{i=L,U} \sum_\nu p_\nu^{(i)} X_\nu^{(i,\alpha)} e^{-\frac{2\pi i \nu n}{N}} \tag{S18}$$

so that $A(0) = \sum_{i\nu} p_\nu^{(i)} X_\nu^{(i,\alpha)}/\sqrt{N}$ results from a collective effect of p-amplitudes that add up with a vanishing phase; large separation amplitudes are instead averaged out by the oscillating exponentials. The wider is the distribution of $p_\nu^{(i)}$, the larger $A(0)$ is expected. Using equations (S15) we get

$$A(0) = \frac{G}{N\sqrt{N}} \sum_{i=L,U} \sum_\nu \frac{X_\nu^{(i,\alpha)} X_\nu^{(i,\beta)}}{\left(E - E_\nu^{(p,i)}\right)} \sum_\mu \Lambda_{\nu\mu} e_\mu. \tag{S19}$$

Due to the mismatch between quantum numbers ν and μ the denominator $(E - E_\nu^{(p,i)})$ is not a real pole. However, it plays an important role in the establishing of the bunching, which occurs when $E = E_\rho$ is resonant with the band of non-interacting states $E_\nu^{(p,i)}$ (S9); when $E_\rho < \min\left\{E_\nu^{(p,i)}\right\} = E_{\nu=0}^{(p,i=L)}$ the two-photon wave function looks unperturbed and exhibits plane-wave-like oscillations. This criterium can be used as a good rule of the thumb when deciding on whether a state with a given energy shows bunching or not. It looks like as if excitons talked to each other via virtual excitations – the eigenstates of the non-interacting subsystem. Indeed, the "real" elementary excitations are one-polariton states, while the energies (S9) do not have an independent physical meaning, except as a virtual scattering channel through which excitons interact.

Using the equality

$$C(s) = \langle \gamma_s | \Psi \rangle = \sum_\mu e_\mu g_s(\mu) \tag{S20}$$

following from $|\Psi\rangle$ representation via A, B, C- and p, e-amplitudes, we find that $2e_\mu = \sum_s g_s(\mu) C(s)$. For higher ρ showing bunching, we can approximate the polaritonic C_ρ-amplitudes by closest in energy (with $\mu = \rho - 1/2$) bare exciton-exciton amplitudes (S3) times a normalization coefficient $X_\rho^{(\gamma)}$, which accounts for the presence of finite exciton-photon and photon-photon excitation in the total wave function of ρ-th eigenstate. We then obtain, using orthogonality of g-functions,

$$A_\rho(0) \approx \frac{G X_\rho^{(\gamma)}}{N^{\frac{3}{2}}} \sum_{i=L,U} \sum_\nu \frac{X_\nu^{(i,\alpha)} X_\nu^{(i,\beta)}}{(E_\rho - E_\nu^{(p,i)})} \Lambda_{\nu(\rho-1/2)}. \tag{S21}$$

The numerator of the equation (S21) shows that only those state contribute into $A(0)$, which *simultaneously* have non-vanishing photonic and excitonic amplitudes, which is true only for the strong coupling region. In addition,

the resonant denominator selects those ν-states which are close in energy to E_ρ. Due to strong dispersion of $E_\nu^{(p,L)}$ inherited from its photonic component, only low-k_ν states contribute to this sum. Therefore, the larger part of the Brilloiun zone the strong coupling region occupies, the stronger is the effect of bunching. This explains the increase of the bunching efficiency with the increase of the lattice constant a: For larger a the Brillouin zone of exciton is matched to a flatter part of the photon dispersion, and the strong coupling region is larger. This scale argument explains why in natural solids the kinematic interaction is a negligible effect, while in atomic systems it may lead to a qualitatively different behaviour.

---

[1] In fact, the Schrödinger equation does not define the amplitudes C_{nn}, as the states $|P_nP_n\rangle$ are not in the basis set of two-exciton states. The choice $C_{nn} = 0$ is the most natural and leads to the simplest form of the equations, see Vektaris, G., A new approach to the molecular biexciton theory. J. Chem. Phys. **101**, 3031 (1994).
